# Developmental expression of the alpha-skeletal actin gene

**DOI:** 10.1186/1471-2148-8-166

**Published:** 2008-06-02

**Authors:** Laura D Bertola, Elisabeth B Ott, Sander Griepsma, Freek J Vonk, Christoph P Bagowski

**Affiliations:** 1 Institute of Biology, Department of Integrative Zoology University of Leiden, 2333 AL Leiden, The Netherlands

## Abstract

**Background:**

Actin is a cytoskeletal protein which exerts a broad range of functions in almost all eukaryotic cells. In higher vertebrates, six primary actin isoforms can be distinguished: alpha-skeletal, alpha-cardiac, alpha-smooth muscle, gamma-smooth muscle, beta-cytoplasmic and gamma-cytoplasmic isoactin. Expression of these actin isoforms during vertebrate development is highly regulated in a temporal and tissue-specific manner, but the mechanisms and the specific differences are currently not well understood. All members of the actin multigene family are highly conserved, suggesting that there is a high selective pressure on these proteins.

**Results:**

We present here a model for the evolution of the genomic organization of alpha-skeletal actin and by molecular modeling, illustrate the structural differences of actin proteins of different phyla. We further describe and compare alpha-skeletal actin expression in two developmental stages of five vertebrate species (mouse, chicken, snake, salamander and fish). Our findings confirm that alpha-skeletal actin is expressed in skeletal muscle and in the heart of all five species. In addition, we identify many novel non-muscular expression domains including several in the central nervous system.

**Conclusion:**

Our results show that the high sequence homology of alpha-skeletal actins is reflected by similarities of their 3 dimensional protein structures, as well as by conserved gene expression patterns during vertebrate development. Nonetheless, we find here important differences in 3D structures, in gene architectures and identify novel expression domains for this structural and functional important gene.

## Background

### The actin multigene family

Actin is a cytoskeletal protein that is ubiquitously expressed in many eukaryotic cells. Examples for actin functions include maintenance of the cytoskeleton, cell motility and muscle contraction. Several studies have further shown the importance of actin in a number of cellular processes, e.g. gene transcription and chromosome morphology [1], control of the cell cycle [2], modulation of a variety of membrane responses [3,4], translation of several mRNA species [5,6] and modulation of enzyme activity and localization within the cell [7-11].

Six primary actin isoforms have been identified in higher vertebrates, being alpha-skeletal (ACTA1), alpha-cardiac (ACTC1), alpha-smooth muscle (ACTA2), gamma-smooth muscle (ACTG2), beta-cytoplasmic (ACTB) and gamma-cytoplasmic isoactin (ACTG1) [12] (Additonal file 2). Actins can be classified in three pairs: two isoforms expressed in striated muscle (skeletal and cardiac tissue), two isoforms from smooth muscle (alpha-smooth muscle predominately in vascular tissue and γ-smooth muscle in the gastrointestinal and genital tracts) and two cytoplasmic isoforms [13]. Later studies have reported additional actin isoforms in higher vertebrates [14-16].

### The evolution of the actin multigene family

Cytoplasmic actins in vertebrates resemble actins present in various amoebas, yeast and slime molds [17-21]. Invertebrate muscle actins are more closely related to vertebrate cytoplasmic actins than to vertebrate muscle actin isoforms [22]. Actin isoforms specific for striated muscle tissue first evolved in primitive chordates [22]. Urochordates and lampreys still express an alpha-cardiac-like isoform in their primitive muscles. At the level of early amphibians or stem reptiles this gene probably duplicated, which resulted in an alpha-skeletal and a modern alpha-cardiac isoactin [22]. The smooth muscle isoactins are believed to have evolved during later development of warm-blooded vertebrates [23] and likely originated from an early skeletal muscle actin [22]. Altogether, over 30 different actins have been characterized from various muscle sources, some of them having a very specialized role [24].

The actin multigene family is highly conserved. The sequences of alpha-skeletal and alpha-cardiac actin differ by only four amino acids over a total of 375 residues in cow [13,25]. This is among the highest conservation found in vertebrate actins [26]. Skeletal alpha-actin differs eight amino acids from the alpha-smooth muscle isoform and six amino acids from the γ-smooth muscle isoform in *Bos taurus*. In comparison to the non-muscle actin isoforms, 24 and 25 differences in the amino acid sequence are found for γ- and β-cytoplasmic actin respectively [13]. These differences are mainly located at the amino terminus of the protein [13]. Being the site of most actin-myosin interactions, it has an important role in cytoarchitecture and other protein-protein interactions.

The high sequence conservation between the two striated muscle isoactins is also apparent in their gene structures. Skeletal alpha-actin and alpha-cardiac actin are interrupted by five introns on corresponding locations [23,27,28]. Even non-coding regions show striking similarity between the two genes and between different species [23,29]. Two large segments that constitute most of the 3' untranslated regions (UTR) show a similarity of 92% and 85% between the human and the rat alpha-skeletal actin gene [30]. On average, sequences of rat/human gene pairs show only 37–45% similarity in the 3' UTR. Based on the early separation of the two genes, this high similarity indicates a high selective pressure to conserve the sequence. These conserved UTR's may affect the stability, translational capacity and the intracellular location of the transcripts, thereby further regulating the expression of the protein.

### The expression of actin isoforms

The expression of the different actin isoforms is developmentally regulated in a temporal and tissue-specific manner. In general, the cytoplasmic actins are ubiquitously expressed in all cell types and throughout development. The other two pairs of actin isoforms have been shown to be expressed in striated (cardiac and skeletal actin) and smooth muscle (two isoforms of smooth muscle actin) [13,31].

Earlier studies showed that the striated and smooth muscle pairs are coexpressed during development [32,33]. This coexpression is not very surprising, since there is also a high conservation in the regulatory sequences of these genes [34].

Cardiac alpha-actin, which is the main actin isoform in the adult heart, has also been shown to be the predominant form in early muscle development (in mouse, human and most cultured cell lines) [33,35,36]. It has been hypothesized that this coordinated co-expression occurs to facilitate rapid accumulation of these proteins [37]. In later development the expression of alpha-cardiac actin is downregulated and alpha-skeletal actin becomes the dominant isoform in the adult tissue, accounting for more than 95% of the total striated muscle actin isoforms [30,37-39].

Skeletal alpha-actin is also expressed in the mammalian heart tissue [30,40]. During the late stage of fetal development alpha-cardiac actin becomes the main isoform, but alpha-skeletal actin might still make up for almost half of the total striated muscle actin content [32]. Similar results have been shown in the chicken heart [41]. In somite development of the amphibian *Xenopus laevis *embryos also both alpha-skeletal and alpha-cardiac actin increase in content in a coordinate manner [42]. However, in amphibians there appear to be at least three distinct isoforms expressed in striated muscle[43,44] and only one isoform expressed in smooth muscle [22].

This developmental paradigm has been further complicated by studies which have demonstrated that alpha-smooth muscle isoactin is also expressed during early cardiac and skeletal muscle development in a variety of myofibroblast-like cells [34,45-48]. So far no significant evidence has been found to answer the controversial question whether this co-expression reflects a truly regulated expression or rather is a result from a persistent "leakage" from the initial high expression during early myogenesis. There might also be "leakage" because of partially diverged regulatory elements, resulting in cross-responsiveness on cellular or environmental stimuli. Based on the high sequence similarity and the absence of pathology in a mutant mouse model that expresses high alpha-skeletal actin in the heart, it is hypothesized that at least these two actin isoforms are functionally redundant [49]. The function of these two actins is likely more related to their differential regulation in space and time than to their protein coding information [49].

Although several studies in the past have elucidated the specific expression pattern of alpha-skeletal actin in a model organism, no comparison of different species has been made so far. In this study we compare alpha-skeletal actin expression of five vertebrate species, in at least two distinct developmental. We identified here several novel non-muscular expression domains of alpha-skeletal actin. Furthermore, we compared the sequences, gene architectures and 3D protein models of several species to shed light on the evolutionary history of alpha-skeletal actin.

## Results

### Phylogenetic analysis

**Figure 1:**
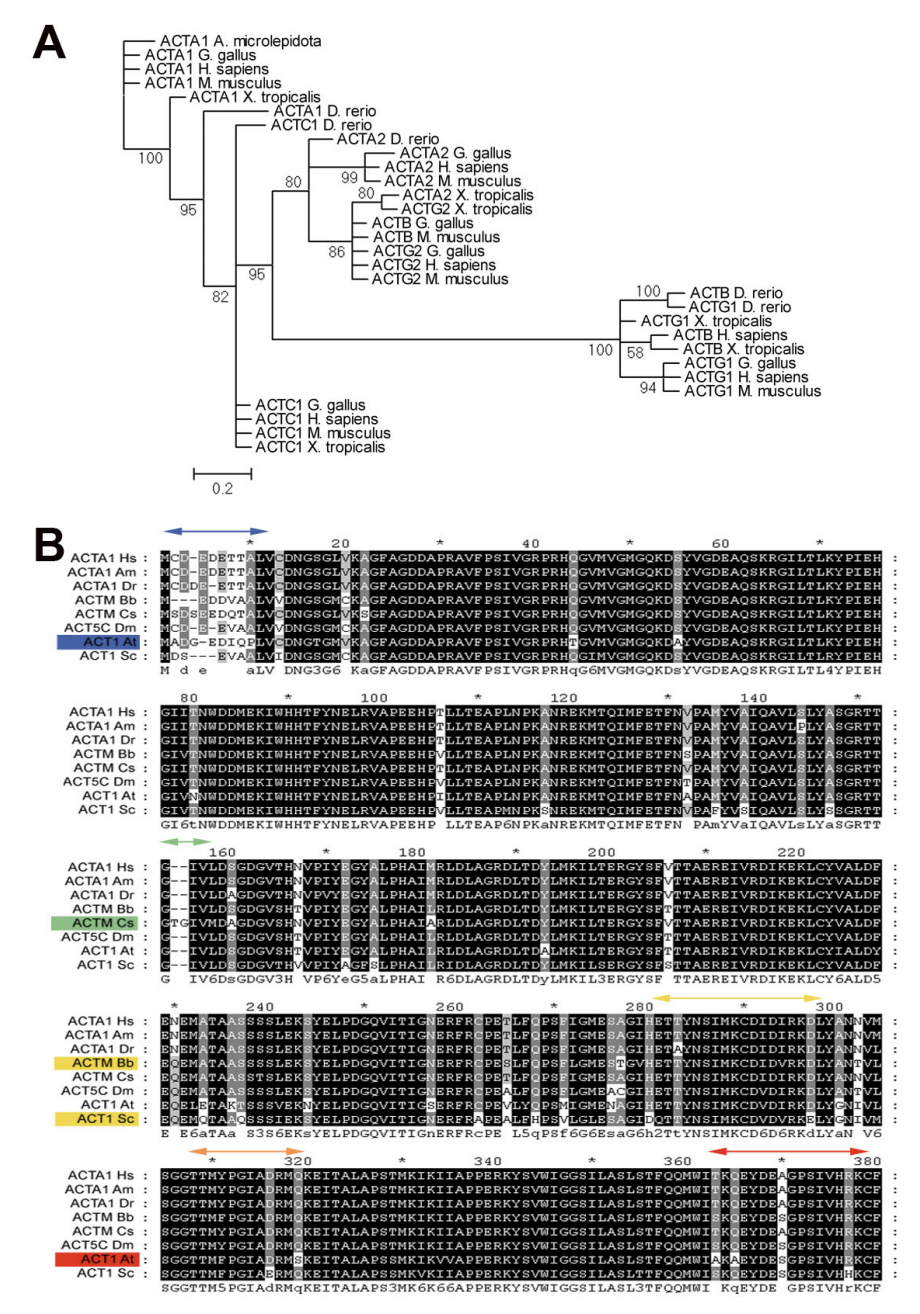
**Phylogenetic tree and amino acid alignment**. **(A) **Shown is the phylogenetic analysis of known actin isoforms from selected vertebrates numbers give the percentages for the Bayesian posterior probability. The accession numbers of the sequences used in this analysis are given in Additional file 1 and additional phylogenetic trees are given in Additional file 3. **(B) **The alignment of the amino acid sequences of the selected actins of human (Hs), snake (Am), zebrafish (Dr), branchiostoma (Bb), urochordate (Cs), fly (Dm), plant (At) and yeast (Sc) is showing the strong conservation of the sequence. The darker shading is used to indicate the more conserved locations. The colored arrows highlight the main sites which show a difference in the 3D protein model (Figure 3). The corresponding coloring of the protein and species name highlights which species show this difference in our 3D protein models.

The sequences of all known actins of selected vertebrate species and of muscle-like actins of a number of other species, covering a broad range of evolutionary history, were extracted from GenBank (Supplemental Table 1). For our phylogenetic analysis, we selected alpha-skeletal actins in several different species, or where that was not possible, an isoform most similar to alpha-skeletal actin by direct sequence comparison. A non-rooted tree is shown in Figure 1 and an additional phylogenetic analysis using yeast actin 1 (ACT1) as an outgroup is shown in Supplemental Figure 1. Invertebrate muscle actin genes cluster with human cytoplasmic actins, as was also the case in previous phylogenetic studies on actins [49-51]. The first muscle actin is found in Deuterostomes, illustrated by the clustering of the sea urchin *S. purpuratus *with the other muscle actins. Actins specific for striated muscle cells are first expressed in early chordates, confirming findings in earlier studies [22]. The alpha-skeletal actins of vertebrates group together, except for the alpha-skeletal actin of zebrafish, which clusters with the actin of the urochordate *Ciona savignyi*. In earlier studies it was already shown that the real alpha-skeletal actins evolved in early amphibians or stem reptiles, explaining why the zebrafish actin is not found on the same branch as the other vertebrates [22].

Interestingly, the sequence of the urochordate *C. savignyi *is closer related to the vertebrate alpha-skeletal actins than the sequence of the branchiostoma *B. belcheri*. A possible explanation might be that the used branchiostoma sequence is not the expected alpha-skeletal actin, although it was identified as the most homologues gene when searched with alpha-skeletal actin sequences of different species.

### Sequence comparison and evolution of genomic architectures

The alignment of the amino acid sequence shows extremely high conservation of the protein in a broad range of species (Figure 1B). Most of the differences are located in the N-terminus of the protein, as has already been shown in a previous study [13]. The shading shows the mode of conservation, the darker blocks being more conserved. In detail black background, conserved in 100% of sequences (8 of 8), dark grey background conserved in more than 70% of sequences, light grey background conserved in more than 60% of sequences and white background below 60% of sequences.

**Figure 2:**
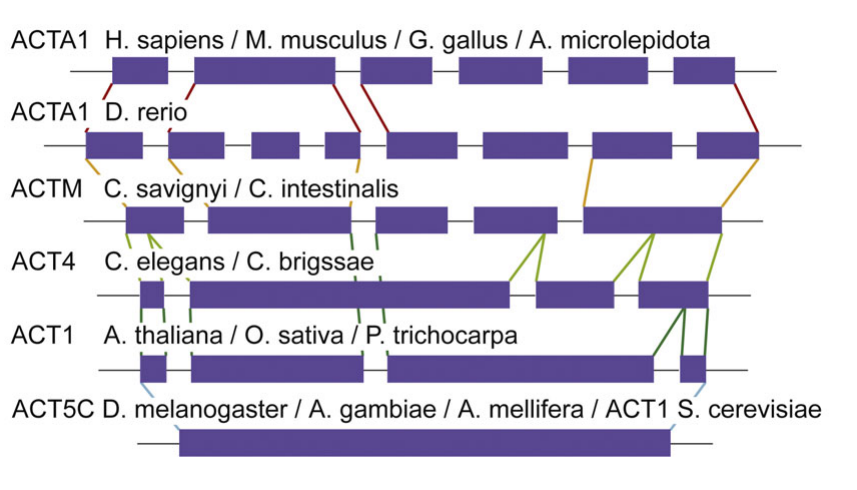
**Comparison of the genomic architecture**. A comparison of the genomic architecture, showing the exons (on scale) and the introns (not on scale). Colored lines were added to indicate the boundaries of the exons. The asterisk marks the intron that has been conserved from plants to higher chordates, but has been lost in insects and nematodes. We have identified the intron-exon boundaries of the alpha-skeletal actin genes in several vertebrates (*Homo sapiens, Mus musculus, Gallus gallus, Atractaspis microlepidota, Xenopus tropicalis, Danio rerio*), chordates (*Ciona savignyi, Ciona intestinalis*), nematodes (*Caenorhabditis elegans, Caenorhabditis briggsae*), insects (*Drosophila melanogaster, Drosophila pseudoobscura, Anopheles gambiae, Aedes aegypti, Culex pipiens, Apis mellifera*), plants (*Arabidopsis thaliana, Oryza sativa, Populus trichocarpa*) and yeast (*Saccharomyces cerevisia*). All insects show a single exon and all vertebrates except the zebrafish *(Danio rerio) *show the same exon/intron boundaries as depicted for humans.

We have identified here the intron-exon boundaries of the alpha-skeletal actin genes in several vertebrates (*Homo sapiens, Mus musculus*, *Gallus gallus*, *Atractaspis microlepidota*, *Xenopus tropicalis*, *Danio rerio*), chordates (*Ciona savignyi*, *Ciona intestinalis*), nematodes (*Caenorhabditis elegans*,*Caenorhabditis briggsae*), insects (*Drosophila melanogaster*, *Drosophila pseudoobscura*, *Anopheles gambiae*, *Aedes aegypti*, *Culex pipiens*, *Apis mellifera*), plants (*Arabidopsis thaliana*, *Oryza sativa*, *Populus trichocarpa*) and yeast (*Saccharomyces cerevisia*). We compare the genomic organization of these alpha-skeletal actin genes and derive an evolutionary model for the development of their gene architecture (Figure 2).

The yeast gene consists of one exon, which is also the case for the studied insect actin genes. In plants and nematodes the gene consists of four exons, but only the first exon shares the same boundaries in these two groups. Surprisingly, the intron between the second and third exon in plants, is located on the same site as the intron in the *Ciona *species. The other exon boundaries differ from plants and nematodes. The last exon in *Ciona *is split in two separate exons in the vertebrates. The zebrafish gene shows another split of the second exon in three separate pieces. The gene architectures of the other vertebrate alpha-skeletal actin genes are even more conserved. The genes in human, mouse, chicken, snake and frog have the same intron-exon boundaries, differing only in the length of the untranslated regions.

In summary, we can show here that in plants, like *Arabidopsis thaliana*,*Oryza sativa *and *Populus trichocarpa*, 4 exons were present. In the course of evolution some species like the studied insect species and also yeast have lost all introns within their alpha-skeletal actin gene. In the nematodes,*Caenorhabditis elegans *and *Caenorhabditis briggsae*, the second intron was lost and exon 2 and 3 fused. However this intron as seen in our evolutionary model in Figure 2 has been conserved from plants to higher chordates. These results show that the genomic organization of the alpha-skeletal actin differs significantly between species.

### Molecular modeling

**Figure 3:**
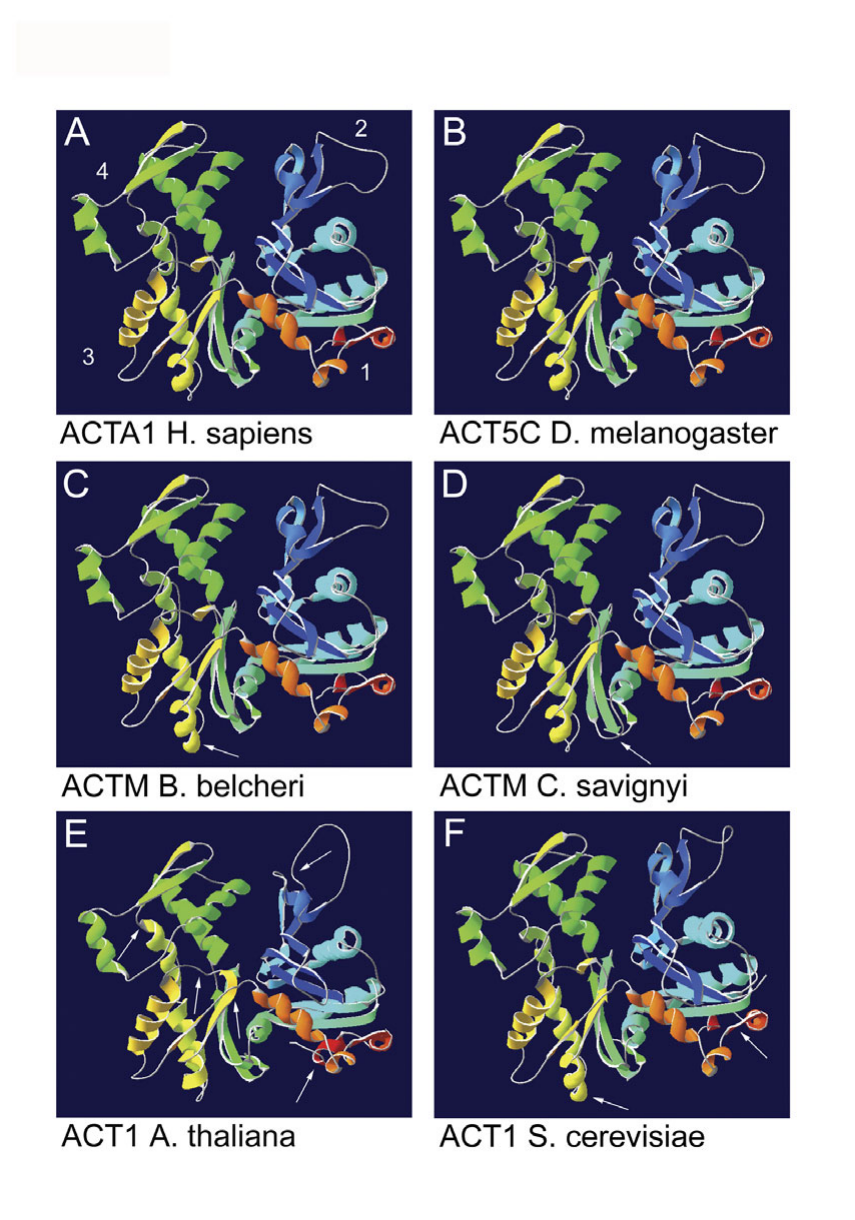
**3D protein models**. 3D protein models of human alpha-skeletal actin (A) and corresponding actins from fly (B), urochordate (C), branchiostoma (D), plant (E) and yeast (F). The secondary structures at the amino terminus of the protein are indicated by dark blue shading and the secondary structures at the C-terminus are colored red. The numbers indicate the subunits of the protein. White arrows point out the main differences between the models. The sites of these differences are highlighted with corresponding coloring in Figure 1.

We modelled here the 3 dimensional protein structures of 19 different actins (see Supplemental Table 1) and show the 6 actins for which structural differences were found (Figure 3). The molecular models of all vertebrate species, *S. purpuratus*, *D. melanogaster *and *C. elegans *do not show a structural difference when compared to each other (data not shown). Additionally, we have found no visible difference between the models of the six different human actins (data not shown).

**Table 1:** The expression of alpha-skeletal actin in different vertebrate species.

Organism	Gene expression	Reference (Method)
Mouse	somites, forelimb buds, hindlimb buds, heart, **liver**, **branchial arches**,**mandible**,**lens**, **otic vesicle**,**brain regions**	This study (*in situ *hybridization)
Mouse/Rat	somites, forelimb buds, hindlimb buds, heart, trunk, head and paraxial mesenchym, cephalic musculature, intraembryonic coelom	Sassoon *et al*. (1988) [38] (*in situ *hybridization) Lyons *et al*. (1991) [53] (*in situ *hybridization and alpha-skeletal actin specific polyclonal antibody) McHugh *et al*. (1991) [48] (Northern blot) Asante *et al*. (1994) [69] (alpha-skeletal actin – Lac Z transgene)
Chicken	somites, forelimb buds, hindlimb buds, heart, **liver**, cephalic musculature, **branchial arches**, **lens**, **otic vesicle**,**nasal pituitary**, **brain regions**	This study (*in situ *hybridization)
	somites, skeletal muscle (limb buds), heart	Hayward & Schwartz (1986) [33, 34] (cell culture hybridization) Ruzicka & Schwartz (1988) [34] (*in situ *hybridization)
Snake	**somites**, **heart**, **liver**, **cephalic musculature**, **branchial arches**,**proliverative cell layer**, **otic vesicle**,**nasal pituitary**, **brain regions**	This study (*in situ *hybridization)
Salamander	somites, **heart**	This study (*in situ *hybridization)
Frog	somites	Mohun *et al*. (1984) [42] (gene-specific probe) Boardman *et al*. (1992) [70] (actin – β-globin fusion gene)
Zebrafish	blastomeres, EVL, somites, pectoral fin musculature (not shown), heart, **liver**, cephalic musculature, **branchial arches**,**jaw musculature**,**lens**, **proliverative cell layer**,**cerebellum**,**epiphysis**,**midbrain**, **hindbrain**,**midcerebral vein**	This study (in situ hybridization)
	blastomeres, EVL, paraxial mesoderm, adaxial cell, somites, pectoral fin, musculature, cephalic musculature	Rauch et al. (2003-present) [71] (in situ hybridization) Thisse & Thisse (2004-present) [72] (in situ hybridization) Lee et al. (2006) [73] (in situ hybridization) Lin et al. (2006) [74] (alpha-skeletal actin – RFP transgene) Hinits and Hughes (2007) [75] (in situ hybridization)

For each of the organisms that were used here, we list the expression domains found in this study in the upper row. Newly in this study discovered expression domains are shown in bold. Expressions domains that have been described in previous studies are listed in the second row.

However, we found important structural differences between the other species investigated (Figure 3). The major changes between the other muscle actins in secondary structures are pointed out by arrows. These sites are also mapped on the alignment of the primary protein sequences (Figure 1B). The *B. belcheri *model shows an additional coil in the alpha helix in subdomain 2 (yellow). This is also the case for yeast, which also shows a difference in the structure on the right in subdomain 1 [52]. In *C. savignyi *the beta sheets in the middle of the molecule in subdomain 3 are a little shorter (green). In the *A. thaliana *model the secondary structures in subdomain 2 are shorter (dark blue), whereas the alpha helix in subdomain 4 is longer (yellow-green). The structure in subdomain 1 [52] and the beta sheet in subdomain 3 (yellow) are also longer. Besides, the *A. thaliana *protein seems to miss the short structure in the middle of the molecule in subdomain 3 (yellow), which is present in all the other models. This structure might as well be fused with the beta sheet in subdomain 3 (yellow), explaining why this structure is significantly longer in *A. thaliana*.

Not all differences in the protein model can easily be recognized in the amino acid sequence. The corresponding regions in the alignment are highlighted by colored arrows (Figure 1B). The ultimate configuration of the protein depends on several regions and their allosteric interactions, sometimes a change in the amino acid sequence can have an effect on a distant part of the protein, where the respective amino acid is actually not located. This explains why some regions, which show differences in the model, do not show clear differences in the alignment. At the same time, the alignment shows less conserved regions, which are not detectable as such in the 3D model. The structural differences we describe here can point to important functional differences or affect specific molecular interactions of actins. It is important to note, that the structural models presented here, are based on predictions and that the subtle changes in structure we observe here, are not likely to have an influence on actins major structural functions e.g. filament assembly or myosin binding. Further studies are necessary to delineate these structure/function relationships and to identify novel functions of actin which could be related to them. Here, molecular approaches (e.g. site-directed mutagenesis) or bioinformatic analysis of potential interactors for example, could give better insights on the significance of our findings.

### Expression in mouse (*Mus musculus*)

**Figure 4:**
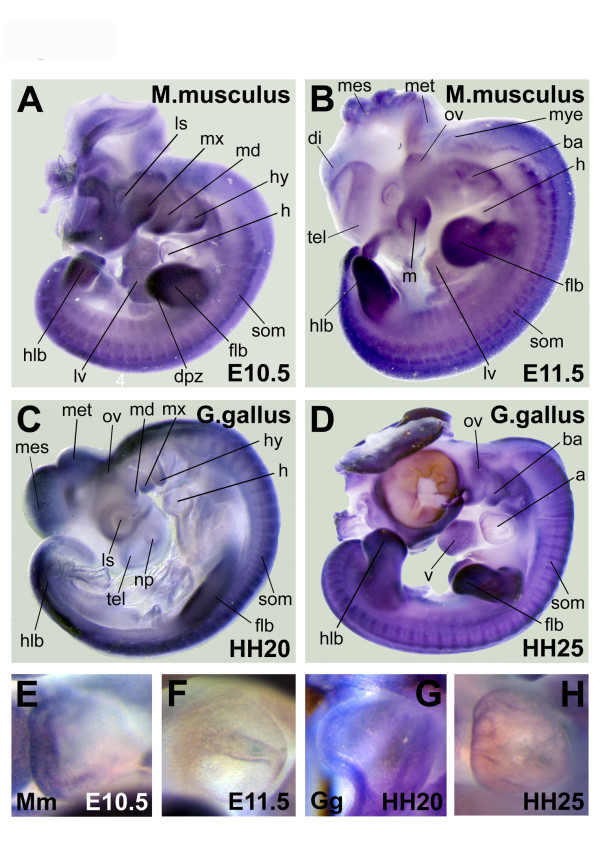
**Expression of alpha-skeletal actin in mouse and chicken embryos**. Results of the *in situ *hybridization on embryos of mouse, stage E10.5 (A) en stage E11.5 (B). Details of the atrium are shown for the early stage embryo (E) and the later stage embryos (F). The same experiment has been conducted on chicken embryos of stage HH20 (C) and HH25 (D). Again details of the atrium are shown for both stages (G, H). Structures with staining are labeled: atrium (a) branchial arches (ba), diencephalon (di), distal progress zone (dpz), fore limb bud (flb), heart (h), hind limb bud (hlb), hyoid arch (hy), lens (ls), liver (lv), mandible (m), mandibular arch (md), maxillary arch (mx), mesencephalon (ms), metencephalon (met), myelencephalon (mye), nasal pituitary (np), otic vesicle (ov), somite (som), telencephalon (tel), ventricle (v).

In the early stage mouse embryo (E10.5) expression seems to be present in several brain areas (Figure 4A). Also the heart, branchial arches (maxillary, mandibular and hyoid arch), lens, hind limb bud, forelimb bud, liver and somites are clearly stained. The strongest staining in the limb bud is located at the most distal part, the distal progress zone and the apical ectodermal ridge (AER).

The mouse at stage E11.5 shows staining in the heart, branchial arches, lens, hind limb bud, forelimb bud, liver and somites (Figure 4B). In addition, the otic vesicle and distinct parts of the brain (telencephalon, diencephalon, mesencephalon, metencephalon and myelencephalon) show staining.

Detailed pictures of the atrium are shown for the early stage embryo (Figure 4E) and the late stage embryo (Figure 4F).

### Expression in chicken (*Gallus gallus*)

In the early stage of the chicken embryo (HH20) the hind limb bud and forelimb bud are strongly stained (Figure 4C). The heart, branchial arches (maxillary, mandibular and hyoid arch), otic vesicle, nasal pituitary, lens, and somites are also stained. In addition, parts of the brain (telencephalon, mesencephalon and metencephalon) show expression.

At the older stage of the chicken-embryo (HH25) there is clear staining in the heart (stronger staining in the ventricle), branchial arches, otic vesicle, liver, forelimb bud and hind limb bud (Figure 4D).

Details of the atrium are shown for the embryos of both stages (Figure 4G, Figure 4H).

### Expression in snake (*Elaphe taeniura frisei*)

**Figure 5:**
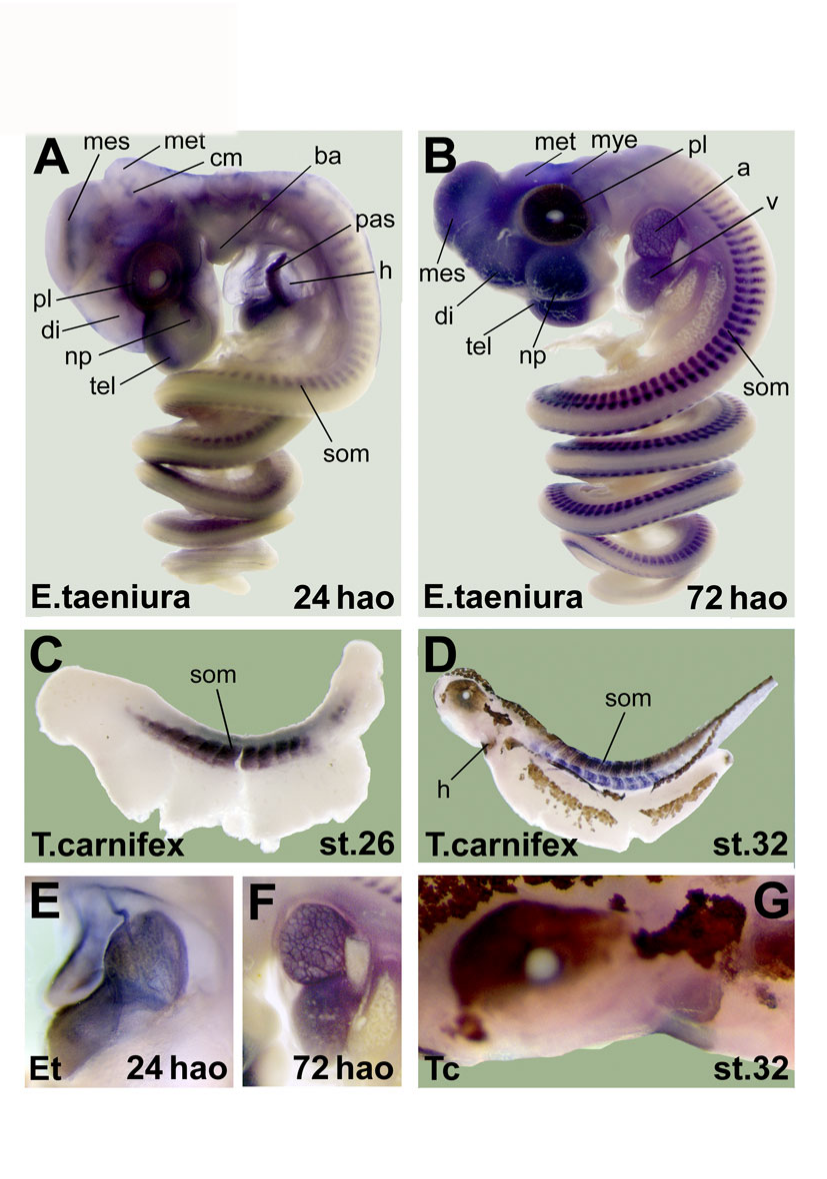
**Expression of alpha-skeletal actin in snake and salamander embryos**. Results of the *in situ *hybridization on snake embryos 24 hours after oviposition (hao) (A) and 72 hao (B). Detailed pictures of the heart are shown for both stages (E, F). Two salamander embryos were also used for an *in situ *hybridization experiment, one staged as st.26 (C) and the older one staged as st.32 (D). A detail of the head and heart region is shown for the oldest embryo (G). The same abbreviations were used for the labeling in Figure 4. Three additional structures were labeled: cephalic musculature (cm), primary atrial septum (pas) and proliferative cell layer (pl).

The youngest snake embryo (24 hao) shows strong expression in the proliferative cell layer (Figure 5A). Furthermore, various brain regions (telencephalon, diencephalon, mesencephalon and metencephalon) and cephalic musculature are stained. Expression in the heart, branchial arches, nasal pituitary and somites is visible as well. Within the heart the strongest staining is located in the primary atrial septum.

In the older snake embryo (72 hao) the proliferative cell layer, heart (again mainly in the ventricle), brain regions (telencephalon, diencephalon, mesencephalon, metencephalon, myelencephalon), nasal pituitary and somites show clear expression (Figure 5B).

Detailed pictures of the heart are shown for the two embryos (Figure 5E, Figure 5F).

### Expression in salamander (*Triturus carnifex*)

The youngest salamander embryo (st.26) only shows staining in the somites (Figure 5C). In the older embryo (st.32) (Figure 5D) there is also expression in the heart. Pigmentation is visible throughout the embryo. This is clearly visible in the detailed picture of the head and heart region (Figure 5G).

### Expression in zebrafish (*Danio rerio*)

**Figure 6:**
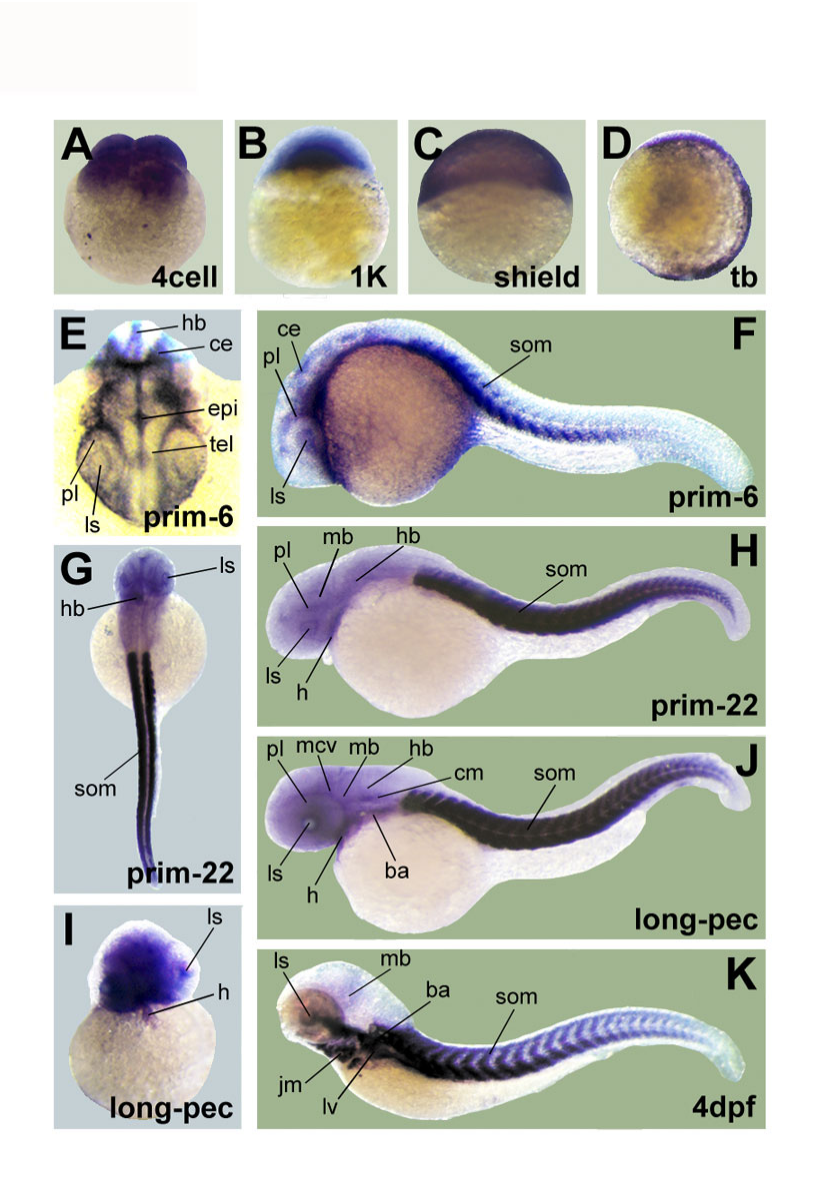
**Expression of alpha-skeletal actin in zebrafish embryos**. Results for whole mount *in situ *hybridization of zebrafish embryos at several stages of development: 4cell (A), 1 K (B), shield (C), tail bud (D), prim-6 (F), prim-22 (H), long-pec (J) and 4 dpf (K). Detailed pictures of dorsal view of the head in prim-6 (E), a dorsal overview picture in prim-22 (G) and a frontal view of the head in long-pec (I) are added. The same abbreviations were used for the labeling as in Figure 4 and Figure 5. Six additional structures were labeled: cerebellum (ce), epiphysis (epi), hindbrain (hb), jaw musculature (jm), midcerebral vein (mcv) and midbrain (mb).

As early as the 4cell stage (Figure 6A), there is staining in the blastomeres. In the 1 K stage the embryo shows expression in the germinal plate (Figure 6B). At the shield stage (Figure 6C) and the tail bud stage (Figure 6D) the staining focuses at the area where the somites will develop. At prim-6 (Figure 6F) there is clear expression at the somites, at the lens and in the proliferative cell layer. There is also some expression in the head region. A detail from the head (Figure 6E) additionally shows staining in the hindbrain, cerebellum, epiphysis and telencephalon. At the prim-22 stage (Figure 1G, Figure 6H) the somites are strongly stained. The lens and proliferative cell layer are a bit weaker stained. Staining is also visible in the midbrain, hindbrain and heart. At the next stage, long-pec (Figure 1I, Figure 1J) there is high expression in the somites, heart, hindbrain, lens, proliferative cell layer, branchial arches, cephalic musculature, midbrain, hindbrain and the midcerebral vein. The last stage, 4 dpf (Figure 1K) shows a strong staining, especially in the branchial arches, jaw musculature, lens, midbrain, liver and somites.

### Comparison of expression patterns

In this study all used embryos displayed clear staining in the somites. Except for the early stage salamander embryo (st.26), all embryos (including the st.32 salamander embryo) showed also expression in the heart. Table 1 gives an overview of expression domains of alpha-skeletal actin in several vertebrate species found in this study. In addition we summarize in Table 1 the findings of other studies on alpha-skeletal actin gene expression. In general, staining is detected in regions which are bound to give rise to skeletal muscle. This is illustrated by the strong staining in the somites and limb buds. We also show here staining in a number of muscles in the head and neck, which also has been described by other studies in rodents and zebrafish [53]. In addition we show that the expression is specifically strong in the branchial arches, which will give rise to a number of facial muscles and muscles that are associated with the larynx and pharynx. All current studies in mouse or chicken have detected expression in the heart as well but expression we show here in the zebrafish heart has not been described before. Here, we also discover alpha-skeletal actin expression in the liver of all species except the salamander. Furthermore, staining in the eye, otic vesicle, nasal pituitary and brain regions has not previously been described.

We can not entirely rule out that there was no crossreactivity of the probe with other isoactins. However, hybridization conditions were very stringent and the fact that most other actins are expressed ubiquitously and that we do not see ubiquitous staining, argues against this. Still, crossreactivity with cardiac actin is not ruled out by this argument but here other studies also have shown that alpha-skeletal actin is weakly expressed in the vertebrate heart.

## Discussion

In this study, we have compared the gene expression patterns of alpha-skeletal actin in five different vertebrate species and describe several novel expression domains for this functional and structural important gene. Because of the high conservation of the actin multigene family, a single probe could be used for the in situ hybridization. This proved to be a useful method when comparing expression patterns of the alpha-skeletal actin gene in these vertebrate species. An important question remains still, how the expression patterns of alpha-skeletal actin and the other actin isoforms are regulated. Here, results from experiments on differentiated, cultured cells have often differed from in vivo studies [33]. Factors such as innervation, growth factors and mitogens, oncogenes, cell density, hormones, mechanical and biological stress and cell-cell interactions may all contribute to the regulation of this multigene family [54-56].

Promoter regions of different actins have been extensively analyzed. So far it is clear that interaction of multiple cis-acting and trans-acting regulatory elements are involved [57,58]. However, the sequences and factors that interact with these regions and confer to positive and/or negative transcriptional regulation are poorly understood. Additionally, a variety of post-transcriptional modifications contribute to the regulation of actin protein expression [59-61]. Future studies should shed more light on these regulatory mechanisms and actin functions. Here, a comparative evolutionary analysis of regulatory elements should help in unraveling some of the signaling pathways and transcriptional networks responsible.

## Conclusion

The phylogenetic analysis, the alignment, the comparison of the genomic architecture and the 3D protein models we show here reveal extreme high conservation of alpha-skeletal actins. The high homology between higher vertebrate actins and muscle-like actins in plant and yeast is striking when looked at the evolutionary distance. Furthermore, alpha-skeletal actin expression patterns show strong conservation in evolution and are very similar in all tested vertebrate species. Interestingly, we find many novel expression domains of alpha-skeletal actin including several in non-muscular tissues and organs.

Despite all observed similarities, we show here also important structural and other differences between alpha-skeletal actins of different species. Our evolutionary model illustrates that the genomic organization of the alpha-skeletal actin differs significantly between species. Insects and yeast for example have lost all introns within their alpha-skeletallike actin genes.

## Methods

### Sequence alignment and phylogenetic analysis

Sequences of all known actins in human and one alpha-skeletal actin isoform, or the closest found homologue in several other species were taken from GenBank (see Additional file 1 for all accession numbers). In other vertebrate species the alpha-skeletal actin was selected. The actins from chordates, invertebrates, plant and yeast were selected by using the sequence most similar to the alpha-skeletal actin isoform. The sequences were manually aligned with ClustalX [62] and subsequently processed with Gblocks, which heuristically removes poorly conserved segments in the alignment in order to enhance the phylogenetic signal [63]. Alignments were manually refined in GeneDoc where necessary. However, the strong conservation within the actin genes made this hardly necessary, except for the N-terminal start regions. Subsequently, the alignments were used to generate the phylogenetic trees employing MrBayes [52] version 3.1. Bayesian trees were generated with MrBayes, with amino acid substitution set to mixed (hence reducing assumption prior to analysis). Rate variation across sites was modeled with a four rate gamma distribution and invariant sites, while the MCMC search itself was continued for 1.000.000 generations, sampled every 100 generations, and 2500 trees were discarded as burnin.

The sequences were also used to model the associated proteins with the automated comparative protein modeling server, Swiss Model. A detailed description is given in one of our previous studies [64].

### Animal care and handling

Animals were handled in compliance with local animal care regulations and standard protocols of the Netherlands. Zebrafish (*Danio rerio*) (Tuebingen line, AlB strain (Albinos) were kept at 28°C in aquaria with day/night light cycles (10 h dark versus 14 h light periods). The developing embryos were kept in an incubator at constant temperature of 28°C. When the required developmental stage was reached, embryos were fixed in 4% PFA. We used six developmental stages for zebrafish: 4cell, 1 K (3 hours post fertilization), shield (6 hpf), tail bud (9 hpf), prim-6 (25 hpf), prim-22 (35 hpf), long-pec (48 hpf) and 4 days post fertilization (staging according to [65]). For mouse (*Mus musculus*), chicken (*Gallus gallus*), snake (*Elaphe taeniura friesei*) and salamander (*Triturus carnifex*) embryos two developmental stages were used for ISH. The stages used were: E10.5 and E11.5 for mouse, HH20 and HH25 for chicken, 24 hours after oviposition (hao) and 72 hao for snake (no official staging system is available for this species yet, the stages we used are comparable to HH20 and HH25 in chicken) and st.26 and st.32 for salamander (staging according to [66]). At least two specimen were used for each species.

All embryos were stored in 100% MeOH at -18°C for several days before the *in situ *hybridization was started.

### *In situ *hybridization [7]

We used a full length probe based on the frog (*Xenopus tropicalis*) alpha-skeletal actin gene [BC075427]. Based on a number of pilot experiments, this probe proved to be effective in locating alpha-skeletal actin in other vertebrate species as well. A sequence comparison of ACTA1, ACTA2 and ACTC1 from different species to the Xenopus laevis ACTA1 is shown in additional file 2.

The *in situ *hybridization procedure in zebrafish was adapted from the Thisse protocol [67] and has been previously described [68]. For the *in situ *hybridization of the other embryos the procedure was slightly adjusted. Depending on the size of the embryo 10–20 μg/ml Proteinase K was used. After rinsing and refixation, the embryos were prehybridized for 2–5 h at 70°C in hybridization buffer (50% formamide, 5× SSC, 1% SDS, 50 μg/ml heparin, 500 μg/ml tRNA). With the snake embryos the prehybridization and hybridization steps were accomplished at 67°C. After incubation in the hybridization buffer, the embryos were washed twice in Solution I (50% formamide, 2× SSC, 1% SDS) at 70°C, twice in Solution II (50% formamide, 1× SSC) at 70°C and 3 times in PBST at RT. The embryos were preabsorbed in 10% sheep serum in PBST for 2 hours at room temperature and incubated overnight at 4°C with a preabsorbed anti DIG antibody at a 1/5000 dilution in the blocking. After staining of the embryos, pictures were taken by stereomicroscopy.

## Authors' contributions

LDB wrote manuscript and performed experiments, EBO performed experiments, SG performed some experiments, FJV supplied snake embryos and read the manuscript and CPB designed and supervised experiments and wrote the manuscript.

## Supplementary Material

Additional file 1 Supplemental Table 1. Click here for file

Additional file 2 Supplemental Table 2. Sequence comparison of ACTA1, ACTA2 and ACTC1 from different species to the Xenopus laevis ACTA1. Click here for file

Additional file 3 Supplemental figure 1. Click here for file
