# Supplementary figures and images for: Developmental expression of the alpha-skeletal actin gene
Source: BMC Evol Biol. 2008 Jun 2;8:166. doi: 10.1186/1471-2148-8-166 (PMC2443135; doi:10.1186/1471-2148-8-166)

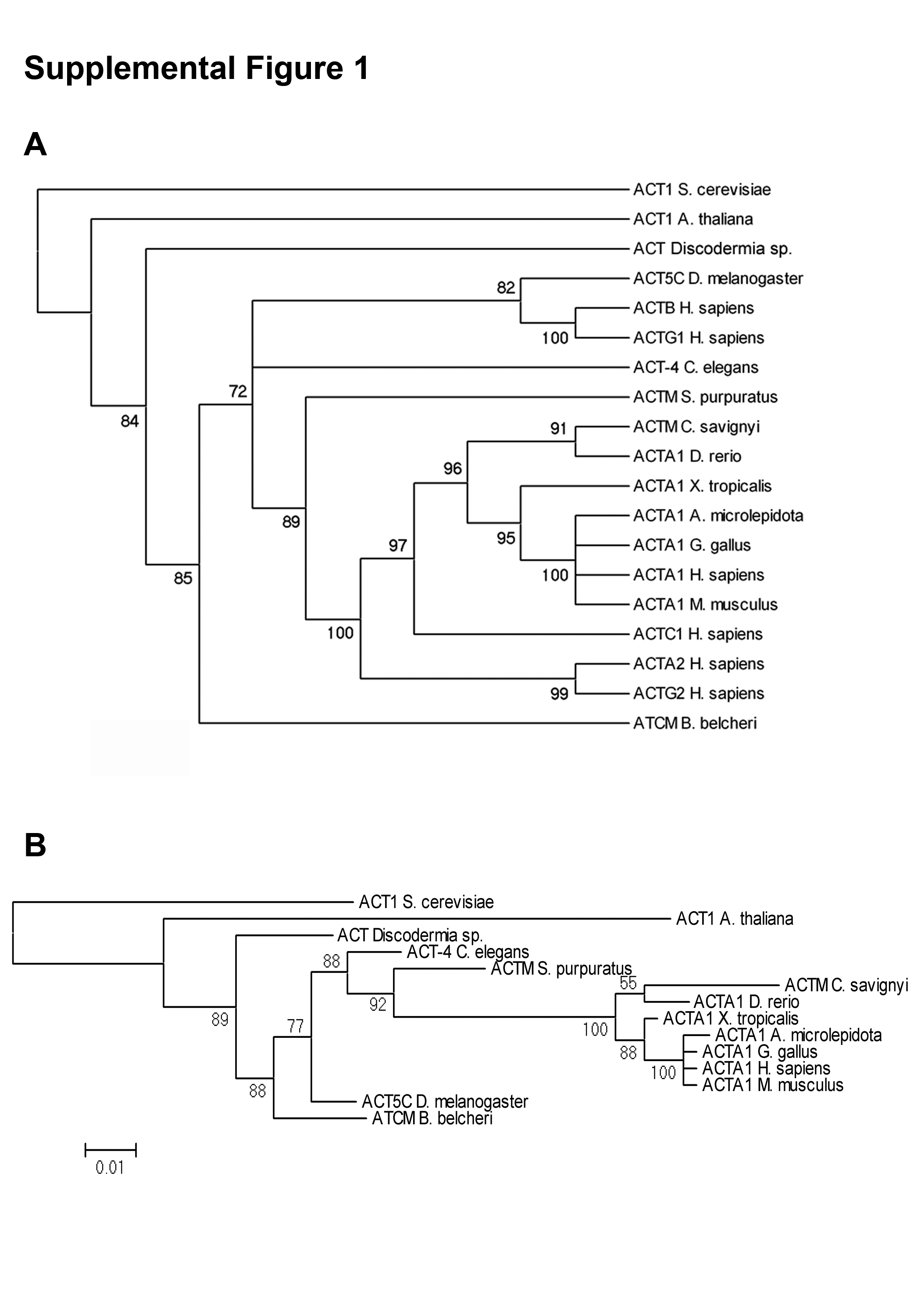

Supplement: Additional file 3 — Supplemental figure 1. [file 1471-2148-8-166-S3.tiff]
